# Ear wax management in primary care: what the busy GP needs to know

**DOI:** 10.3399/bjgp23X732009

**Published:** 2023-01-27

**Authors:** Kevin J Munro, Thomas C Giles, Christine Smith-Howell, Irwin Nazareth

**Affiliations:** NIHR Manchester Biomedical Research Centre, Manchester.; Manchester Centre for Audiology and Deafness, University of Manchester, Manchester.; Trafford Ear Care lead and national trainer, Meadway Health Centre, Manchester University NHS Foundation Trust, Manchester.; Research Department of Primary Care and Population Health, University College London, London.

## INTRODUCTION

Cerumen (or earwax), a self-cleaning agent, protects the outer ear. Sometimes this does not work and wax gets impacted, blocking the ear canal. This is a major reason for primary care consultation. Hearing difficulty due to untreated wax impaction can lead to social isolation and depression.^1^ Yet there is little quality evidence to guide practice for such a common condition. Further, people find it difficult to access NHS earwax services. This article provides new data on symptoms and severity, and reviews management options and patient preferences.

## WHY DOES IT MATTER?

Earwax build-up occurs in anyone but often in older people and those using hearing aids or earbud earphones. Up to 44% of care home residents with dementia also have impacted earwax^2^ and about 2.3 million people/year in the UK have troublesome earwax requiring removal.^3^

Studies investigating earwax removal methods report how much of the tympanic membrane is visualised before and after treatment, but this does not capture the impact of the symptoms. This article reports on a symptom survey completed by 489 patients who attended the Trafford Ear Care Service, Manchester, during 2022. The most common symptom was hearing difficulty (86.5%; 423) with half of those with hearing difficulty reporting additional symptoms, including discomfort, tinnitus, or change in quality of their voice. Before wax removal, the most bothersome symptom was reported to be hearing difficulty (78.3%; 383), impacting on communication, focused listening (for example, TV), and awareness of surroundings. Predicting the effect of impacted wax on an individual is difficult as it depends on the quantity, consistency, and location of the wax within the ear canal.

**Figure 1(a).**
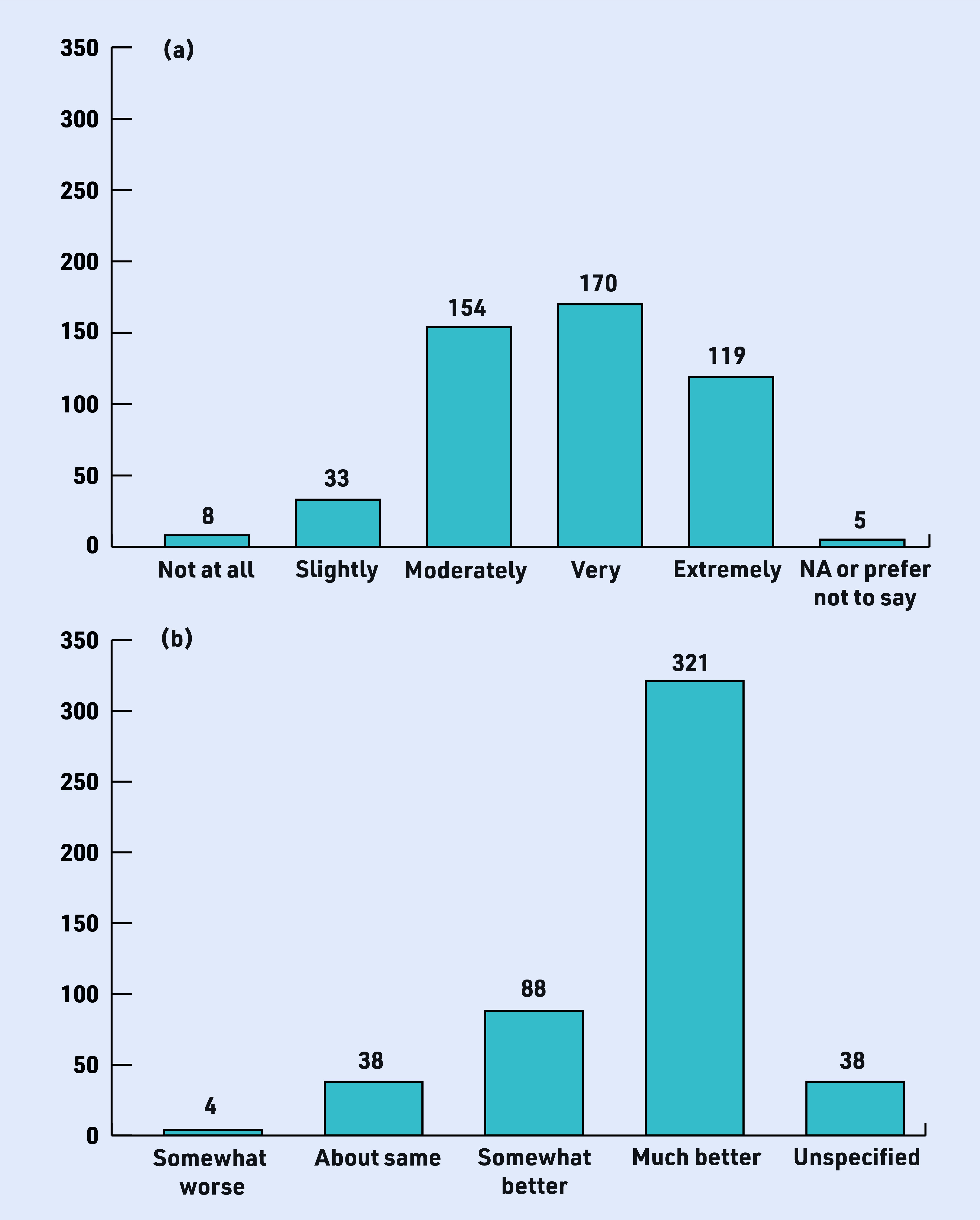
*How bothersome was the earwax before wax removal (*n *= 489); (b) immediate improvement after wax removal (*n *= 489). NA = not applicable.*

Figure 1 shows that more than 90% (*n* = 443) found earwax at least moderately bothersome and 60% very/extremely bothersome before removal (a). After removal, more than 83% (*n* = 409) reported hearing difficulty to be somewhat or much better (b). In some cases, hearing difficulty may have persisted after earwax removal because most patients were older (70–79 years).

## WHY THE CURRENT CONTROVERSY?

The National Institute for Health and Care Excellence (NICE) recommends that earwax removal services should be available in primary care.^1^ However, earwax services are so scarce and non-existent in some locations that user groups led by the Royal National Institute for Deaf People campaign for wider access in primary care.

Controversies raised in parliament and media reports question referral to secondary care (ENT) with long delays, inappropriate use of specialist services, and the need to access private care from non-NHS ‘high street’ hearing aid dispensers. A Healthwatch Oxfordshire survey revealed that adults with earwax required 1–4 NHS visits prior to attending a dewaxing clinic, that time from symptoms to resolution was 3–30 weeks, and that microsuction required additional visits and longer waits.^4^

## WHICH PRE-TREATMENT EARWAX SOFTENER IS BEST?

Pre-treatment drops or sprays soften the impacted wax making it easier for removal. A current concern is that earwax is often untreated with the mistaken belief that self-management with pre-treatment softeners is sufficient. Systematic reviews by both NICE^1^ and Cochrane^5^ fail to conclude which type of ear drop was more effective, or whether water or saline was better or worse than commercially available earwax softeners. NICE recommends pre-treatment softeners for up to 5 days before removal but stop short of recommending any particular product. Studies systematically comparing the benefit of different durations of ear drop use, or the administration of drops versus sprays, are limited.

## WHAT PROCEDURES ARE RECOMMENDED FOR EARWAX REMOVAL AND WHAT DO PATIENTS PREFER?

NICE recommends electronic water irrigation and microsuction for earwax removal (or an alternative such as manual removal). Direct comparison of these two procedures in terms of safety, effectiveness, and costs is lacking. National training courses on wax removal for registered healthcare professionals (nurses, healthcare assistants) are available, with a requirement for an update every 3 years.

Manual water-filled syringes are no longer recommended in the UK because of potential damage to hearing and risk of litigation. Electronic devices that control the flow of low pressured water to flush the earwax from the ear canal are now in use. Contraindications include pre-existing otological conditions (for example, perforated eardrum, grommet, mastoid cavity, infection), presence of foreign body, or previous problems with wax removal. Specialist referrals resulting from complications of irrigation (for example, perforated eardrum) are estimated at 1/1000.^6^ There are anecdotal reports that drying the external ear after irrigation reduces the risk of ear infection. Irrigation is contraindicated in patients with only one functioning ear.

The alternative is to remove wax under direct visualisation using mechanical suction, the method of choice in secondary care. The cost of freestanding operating microscopes for microsuction is impractical in primary care. Lower-cost, portable hand-held video-assisted systems providing magnification and illuminated visualisation of the ear canal are a potential solution for widescale use.^7^ Little is known of the safety of microsuction although there are reports of discomfort and minor bleeding.

**Figure 2.**
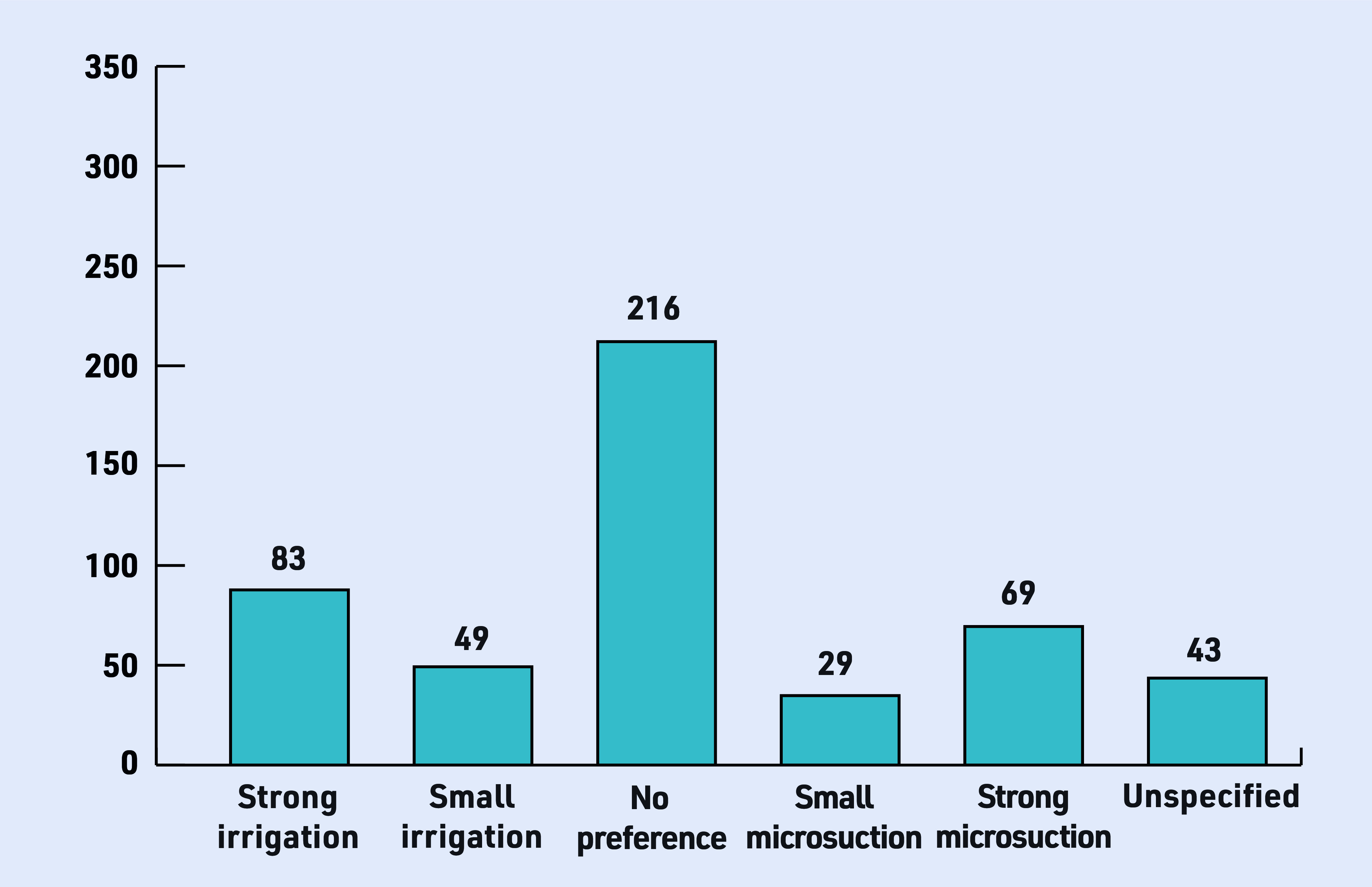
***Preference for microsuction and water irrigation (*** **n *= 489).***

In the service evaluation mentioned above, all patients were offered both treatments and more than two-thirds had no or only a small preference for ether irrigation or microsuction (Figure 2). The proportion with direct experience of both procedures is unknown, but those expressing a preference for irrigation sometimes reported that previous microsuction was painful or noisy whereas those preferring suction report it is less messy compared with irrigation. There is currently no high-quality evidence comparing professionally administered and home-based procedures. Alternative methods of treating earwax such as ear candles are not recommended because these are ineffective.^8^ Also, inserting cotton buds into the ear canal has the potential to damage the ear and cause wax impaction.

A Health Technology Assessment review concluded that further research is required to improve the evidence base such as a randomised control trial (RCT) incorporating an economic evaluation to assess different pathways, different removal methods, and acceptability of the different approaches.^9^

## WHAT PATHWAY?

Groups of practices rather than each individual practice can collaborate as primary care networks to provide a range of services, such as earwax removal. The portable nature of contemporary wax removal equipment is optimal in such a setting and for use in domiciliary visits to care homes. The cost of setting up a video-assisted mobile earwax suction service within a group of practices would involve an initial set-up and training cost-of around £1000, and any ongoing cost associated with rental of mobile equipment. However, the feasibility, clinical and cost effectiveness of this care pathway needs testing in an RCT.

## TAKE-HOME MESSAGE

- A significant number of people fail to get the care they need for earwax removal and there is an urgent need for such a service in primary care.
- Pre-treatment softeners are recommended followed by removal using electronic water irrigation or microsuction.
- The use of modern portable equipment within a primary care network and for use in care homes is a possible approach.
- Further evidence on the delivery of such a service is urgently required.
